# Supplementary material for: Impact of Novel Active Layer-by-Layer Edible Coating on the Qualitative and Biochemical Traits of Minimally Processed ‘Annurca Rossa del Sud’ Apple Fruit
Source: Int J Mol Sci. 2023 May 5;24(9):8315. doi: 10.3390/ijms24098315 (PMC10179199; doi:10.3390/ijms24098315)
Supplement: Supplementary file 1 [file ijms-24-08315-s001.zip › ijms-2344254-supplementary.pdf]

Table S1. Colour parameters (L*: lightness; BI: browning index %) of control (C) and treated with CMC+SA (TA), CMC+SA+CA (TB), CMC+SA+AO (TC) and CMC+SA+CA+AO (TD) fresh cut 'Annurca Rossa del Sud' apple after 0 (T0), 4 (T1), 8 (T2) and 12 days (T3) of cold storage at 4 °C ± 0.5. The results are reported as mean ± standard deviation. Means followed by the same letter do not differ significantly at $P = 0.05$ (Duncan's test).

Colour Parameters	Timing	C	TA	TB	TC	TD
L*	T0	96.22 ± 0.81 l	96.22 ± 0.81 l	96.22 ± 0.81 l	96.22 ± 0.81 l	96.22 ± 0.81 l
	T1	87.72 ± 0.52 i	73.74 ± 0.20 b	71.69 ± 0.23 a	85.92 ± 0.37 h	87.93 ± 0.32 i
	T2	81.42 ± 1.06 f	79.24 ± 1.24 e	77.52 ± 1.50 d	79.26 ± 1.00 e	88.77 ± 0.74 i
	T3	79.80 ± 0.46 e	75.91 ± 1.15 c	79.08 ± 1.03 e	79.77 ± 0.14 e	84.39 ± 0.29 f
a*	T0	-3.44 ± 0.16 bc	-3.44 ± 0.16 bc	-3.44 ± 0.16 bc	-3.44 ± 0.16 bc	-3.44 ± 0.16 bc
	T1	-3.80 ± 0.08 a	-2.58 ± 0.09 d	-3.51 ± 0.19 bc	-3.57 ± 0.04 bc	-3.13 ± 0.10 c
	T2	2.78 ± 0.21 g	1.93 ± 0.05 f	-4.22 ± 0.06 a	-3.55 ± 0.11 bc	-0.87 ± 0.77 e
	T3	8.75 ± 0.20 i	9.77 ± 0.48 l	4.19 ± 0.29 i	-2.46 ± 0.14 d	-2.54 ± 0.13 d
b*	T0	26.77 ± 0.13 b	26.77 ± 0.13 b	26.77 ± 0.13 b	26.77 ± 0.13 b	26.77 ± 0.13 b
	T1	50.52 ± 0.26 h	37.59 ± 0.35 e	24.77 ± 0.24 a	27.64 ± 0.06 b	25.21 ± 0.42 a
	T2	52.55 ± 1.38 i	44.35 ± 1.51 g	37.75 ± 1.09 e	32.10 ± 0.65 c	28.03 ± 0.81 b
	T3	55.74 ± 0.88 l	45.40 ± 0.33 g	39.58 ± 0.92 f	34.84 ± 1.47 d	27.45 ± 0.27 b
BI	T0	0 ± 0 a	0 ± 0 a	0 ± 0 a	0 ± 0 a	0 ± 0 a
	T1	51.97 ± 0.80 i	34.53 ± 0.74 g	16.91 ± 0.74 d	5.77 ± 0.14 c	1.42 ± 0.52 ab
	T2	64.73 ± 5.01 l	42.72 ± 2.29 h	30.30 ± 3.87 f	18.07 ± 1.39 d	4.47 ± 0.80 bc
	T3	81.58 ± 3.51 m	52.58 ± 1.24 i	32.20 ± 0.68 fg	22.02 ± 2.97 e	6.70 ± 0.54 c
